# Effect of increased venous blood flow with acute passive heating on volume and compliance in the calf vein in young adults

**DOI:** 10.14814/phy2.70700

**Published:** 2025-12-09

**Authors:** Yasuhiro Iimura, Anna Oue

**Affiliations:** ^1^ Department of Nutritional Sciences, Faculty of Health and Sports Sciences Toyo University Tokyo Japan

**Keywords:** doppler ultrasound, endurance exercise training, shear rate, venous distensibility, venous occlusion plethysmography

## Abstract

Arterial endothelial function—impairment of which is a risk factor for cardiovascular disease—increases after acute passive heating; however, whether venous properties (volume and compliance) also increase remains unclear. Moreover, it is unknown whether venous congestion induced by proximal thigh cuff inflation and passive heating exerts opposing effects on the venous properties. Therefore, in 21 young healthy adults, we determined the acute effects of passive heating (42°C) of the left leg and concurrent cuff inflation (80–110 mmHg) of the right leg for 40 min on venous properties. Specifically, before (Pre) and 60 min after (Post 60) the single manipulation, the venous volume and compliance in both legs were determined from the change in calf volume during a cuff deflation protocol. After passive heating, the calf venous compliance decreased (Pre, 0.090 ± 0.032 mL/dL/mmHg; Post 60, 0.084 ± 0.031 mL/dL/mmHg; *p* < 0.05), but calf venous volume did not change (Pre, 3.18 ± 1.10 mL/dL; Post 60, 3.01 ± 1.14 mL/dL; *p* > 0.05). While the volume (Pre, 3.32 ± 1.08 mL/dL; Post 60, 3.19 ± 0.86 mL/dL; *p* > 0.05) and compliance (Pre, 0.091 ± 0.028 mL/dL/mmHg; Post 60, 0.092 ± 0.033 mL/dL/mmHg; *p* > 0.05) in calf veins did not alter after thigh cuff inflation. These results suggest that acute passive heating decreases calf venous compliance with no change in calf venous volume, while thigh cuff inflation does not alter volume and compliance in the calf veins in healthy young adults.

## 1 INTRODUCTION

Passive heating such as hot‐water immersion or sauna has been shown to be beneficial interventions that improve cardiovascular disease and hypertension (Brunt, Howard, et al., 2016; Imamura et al., 2001; Kihara et al., 2002; Miyata et al., 2008) as well as the improvement of arterial vascular properties. Indeed, long‐term passive heating and acute passive heating increase endothelial function (Brunt, Howard, et al., 2016; Brunt, Jeckell, et al., 2016; Restaino et al., 2015; Teixeira et al., 2017) and decrease arterial stiffness (Brunt, Howard, et al., 2016; Caldwell et al., 2017; Ogoh et al., 2016; Sugawara & Tomoto, 2021). These effects are thought to be mediated, at least in part, by heat‐induced increased blood flow and shear stress that enhance the bioavailability of endothelial nitric oxide (NO) (Cheng & Macdonald, 2019).

Compared with the arteries, veins have a high compliance and contain approximately two‐thirds of the total blood volume at rest, and small changes in venous tone can modulate venous return and contribute to the maintenance of circulatory homeostasis under various stresses (Rothe, 1983). For instance, during passive heating, venous blood flow increases (Abraham et al., 1994; Ooue et al., 2007). The accompanying shear stress upregulates endothelial nitric oxide synthase (eNOS) mRNA and/or increases NO release (Koller et al., 1998; Noris et al., 1995), and veins dilate in an NO‐dependent manner (Fukaya & Ohhashi, 1996). These hemodynamic and endothelial adjustments promote cutaneous perfusion and thereby enhance both evaporative and non‐evaporative heat loss (Rowell, 1974). Furthermore, the high compliance of the limb veins provides venous capacitance that accommodates greater blood volumes in superficial tissues. Indeed, heat acclimation has been associated with increased venous compliance (Maruyama et al., 2006). These observations suggest that the volume and compliance in calf veins might also increase after acute passive heating; however, to our knowledge, this speculation has not been investigated. Moreover, venous compliance decreases with aging and physical inactivity (Bleeker et al., 2004; Olsen & Länne, 1998; Sielatycki et al., 2011; Young et al., 2006), changes that are believed to be partly related to elevated blood pressure (BP) and the pathogenesis of hypertension (Delaney et al., 2008; Safar & London, 1987). In addition, an association between lower venous compliance and higher BP has been reported in humans (Oue et al., 2020). Therefore, investigating the effect of acute passive heating on the venous properties is important for both thermoregulatory physiology and cardiovascular health.

In contrast to passive heating, cuff inflation of the limb can decrease arterial endothelial function in a pressure‐dependent manner (Thijssen et al., 2009). Reductions in blood flow and shear stress with cuff inflation are linked to increased oxidative stress (Hwang et al., 2003) and endothelin‐1 (Spieker et al., 2003), which can impair endothelial function (Nishiyama et al., 2017). The cuff inflation of the thigh induces venous congestion (Okazaki et al., 2005), elevates venous pressure (Bauer et al., 2002; Halliwill et al., 1999), evokes the venous distension reflex (activating sympathetic nerve activity) (Cui et al., 2011; Williamson et al., 1994), and increases local vasoconstrictors (e.g., endothelin‐1, angiotensin II) (Colombo et al., 2014; Lin et al., 2017). However, the effects of acute cuff inflation–induced venous congestion per se on the volume and compliance in calf veins remain unclear. Moreover, it is unknown whether cuff inflation and passive heating exert opposing effects on the volume and compliance.

By concurrently assessing passive heating and thigh cuff inflation in a within‐participant, contralateral‐limb design, we can provide new insight into how bidirectional changes in blood flow and shear rate (increases and decreases) affect the venous properties. Accordingly, the purpose of this study was to clarify the acute effect of passive heating and cuff inflation on the volume and compliance in calf veins. Previous studies have reported that the increase in arterial blood flow with local passive heating at 42°C for 30 min returned to baseline by 30 min after heating, although the improvement of endothelial function remained for 30–120 min after passive heating (Brunt, Howard, et al., 2016; Brunt, Jeckell, et al., 2016; Chaseling et al., 2023). Therefore, venous properties in our study were also evaluated after a 60 min recovery period following warming. We hypothesized that passive heating would increase venous blood flow and shear rate, thereby enhancing endothelial NO bioavailability—via increased NO release and /or upregulation of NO synthase—and, as a result, the volume and compliance in the veins would increase after acute passive heating. In addition, we hypothesized that cuff inflation would induce venous congestion, elevated venous pressure, reflex activation, and increased local vasoconstrictors, thereby decreasing the volume and compliance in calf veins after acute cuff inflation.

## 2 METHODS

### 2.1 Subjects

Twenty‐one young healthy volunteers (6 females, 15 males) participated in this study (mean ± standard deviation: age, 21 ± 1 years; height, 167.4 ± 5.7 cm; weight, 63.4 ± 12.2 kg; BMI, 22.6 ± 4.0 kg/m^2^; body fat percentage, 21.0% ± 8.2%). According to their medical histories and physical examinations, none of the participants had been diagnosed as having cardiovascular disease, diabetes, insulin resistance, or cardiovascular risk factors, such as hypercholesterolemia or hypertension. The study was approved by the Human Ethics Committee of Toyo University (approval no. TU2021‐033) and was conducted in accordance with the Declaration of Helsinki. All participants provided written informed consent after receiving an explanation of the purpose, procedures, and risks of the study. The participants abstained from caffeine, alcohol, and intense exercise for 24 h before the experiments and from food intake for 2 h beforehand.

### 2.2 Experimental conditions

We set the experimental conditions of passive heating and cuff inflation separately between the left and right legs within a participant. The left leg was submerged up to the calf in water at a constant temperature of 42°C in order to increase popliteal venous blood flow (passive heating leg). In contrast, as a venous congestion condition, a cuff was placed on the right thigh and inflated to 80–110 mmHg in the open air to evoke venous congestion in the lower limb (cuff inflation leg). In addition, the degree of cuff pressure inflation was chosen at the time at which no popliteal venous blood velocity was detected at rest, and this value was decided separately for each participant.

### 2.3 Experimental design

Participants came to our laboratory on three occasions. On the first day, the participants were familiarized with the experimental procedure and equipment. On the second day, we confirmed that popliteal venous blood flow is increased by passive heating in the left leg and that venous congestion in the lower limb occurs by cuff inflation in the right leg. The protocol of calf venous properties before and after a single manipulation was conducted on the third day.

### 2.4 Protocol to measure venous blood flow responses in the passive heating leg and cuff inflation leg

After the participants rested in a sitting position for at least 20 min in a room with the temperature maintained at 26.3°C ± 0.9°C, the blood velocity and diameter of the popliteal vein in both legs were measured as baseline data. Then, we measured the blood velocity and diameter of the popliteal vein in the left leg without passive heating and in the right thigh with inflation to 80–110 mmHg. Finally, the participants immersed their left leg in the warm water at 42°C with simultaneous inflation of the cuff on the right leg to 80–110 mmHg for 40 min. The blood velocity and diameter of the popliteal vein in both legs were measured at between 35 and 40 min of passive heating and cuff inflation.

### 2.5 Protocol of calf venous properties before and after a single manipulation

**FIGURE 1:**
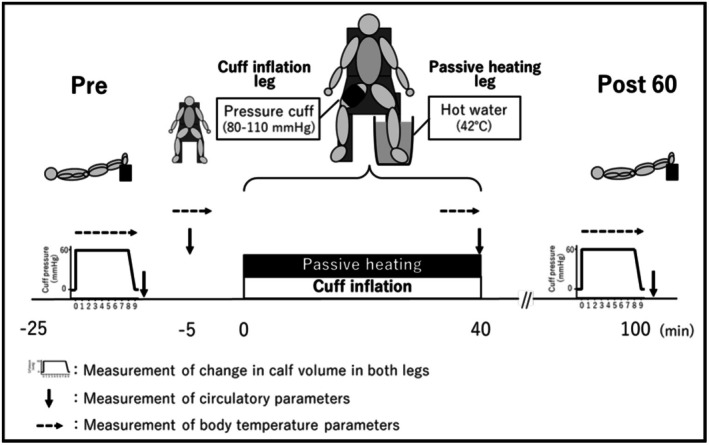
Schematic diagram of the protocol of calf venous properties before and after a single manipulation. Pre, before passive heating and cuff inflation; Post 60, 60 min after passive heating and cuff inflation.

After arriving at our laboratory, participants rested in the supine position for at least 20 min in a room with the temperature maintained at 26.2°C ± 1.0°C. Then, the changes in calf venous volume in both legs were measured by venous occlusion plethysmography (VOP) during a cuff deflation protocol (Halliwill et al., 1999) in order to obtain baseline data (Pre). Following the Pre measurement, the participants rested in a chair for 5 min and then immersed their left leg in warm water in a sitting position while the cuff inflation was applied to the right leg for 40 min. At the end of the 40 min, the participants rested again in the supine position, and the changes in calf venous volume in both legs were measured at 60 min after the passive heating and cuff inflation (Post 60) (Figure 1).

### 2.6 Measurements

#### 2.6.1 Popliteal venous blood flow responses

The blood velocity and diameter of the popliteal vein were measured using duplex‐Doppler ultrasound (LOGIQe; General Electric International Japan Investments B. V., Tokyo, Japan) and a linear‐array probe (L4‐12t‐RS Probe; General Electric International Japan Investments B. V.). The probe was placed over the popliteal vein just proximal to the popliteal fossa. The blood velocity was obtained in duplex mode at a pulsed frequency of 6.3 MHz and corrected with an insonation angle of ≤60°, and diameter signals were set to optimize longitudinal B‐mode images of the lumen/venous wall interface. Sample volume was adjusted to encompass the entire lumen of the vessels without extending beyond the walls, and the cursor was set at the mid vessels. The diameter was manually detected. The popliteal venous blood flow was calculated by the product of the lumen cross‐sectional area [(diameter/2)^2^ × π] and mean blood velocity. In addition, shear rate was calculated as [(4 × mean blood velocity)/diameter] (Carter et al., 2014; Ogoh et al., 2021; Tinken et al., 2009).

#### 2.6.2 Calf venous properties

To measure the changes in calf venous volume in both legs, the venous collecting cuff was wrapped around both thighs and strain gauges were placed on the thickest site of the calf. Then, the collecting cuff was inflated to 60 mmHg for 8 min, after which the cuff pressure was manually reduced at a rate of 1 mmHg/s from 60 to 0 mmHg according to a previously described cuff deflation protocol (Halliwill et al., 1999). Throughout the cuff deflation protocol, the changes in calf venous volume in both legs were measured noninvasively by VOP (EC4; D. E. Hokanson, Bellevue, WA). Because cuff inflation at 60 mmHg evokes fluid filtration, which can lead to volume and compliance overestimation in calf veins, we corrected for the increase in calf venous volume during the cuff deflation protocol caused by fluid filtration, following the method used in a previous study (Skoog et al., 2015). Using the corrected calf venous volume, the relationship between the cuff pressure and the change in calf venous volumes (i.e., the pressure–volume curve) was generated from the data points between 10 and 60 mmHg during the cuff deflation protocol. To avoid any a priori assumption regarding the cuff pressure (P)–venous volume (V) curve and to obtain the physiological venous compliance relationship, venous compliance was calculated as the numerical derivative of each pair of cuff pressure–venous volume data points by the following equation (Freeman et al., 2002).

$${\text{Venous compliance}}_{\mathrm{pi}}=\frac{\left(V_{i}-V_{i-10}\right)}{\left(P_{i}-P_{i-10}\right)}\;\left(\text{where}\;20\leq i\leq 60\right)$$

Venous volume was evaluated as the corrected calf volume value at 8 min after the start of a cuff inflation at 60 mmHg of cuff pressure (Oue et al., 2020). Venous compliance at 20 mmHg of cuff pressure was used as the representative value (Halliwill et al., 1999; Monahan et al., 2001).

#### 2.6.3 Circulatory parameters

Heart rate (HR) was monitored via an HR monitor (T31 coded transmitter; Polar Electro, Kempele, Finland). Systolic BP (SBP) and diastolic BP (DBP) were measured on the upper left arm with the auscultatory method using a sphygmomanometer (Tango M2 Stress Test Monitor; SunTech Medical Inc., Morrisville, NC). Mean arterial blood pressure (MAP) was calculated as follows: (SBP − DBP)/3 + DBP. HR, SBP, and DBP were measured at sitting rest and at 40 min of passive heating and cuff inflation. In addition, HR, SBP, and DBP in supine rest were measured immediately after completion of the cuff deflation protocol at Pre and Post 60 (Figure 1).

#### 2.6.4 Body temperature parameters

During the Protocol of calf venous properties before and after a single manipulation, the local skin temperatures of the thigh, calf, and feet in both the left and right legs were measured using copper‐constantan thermocouples taped to the skin and recorded with a data logger (MX100; Yokogawa, Tokyo, Japan) every second. In both legs, the means of the sitting rest period for 5 min and of the passive heating and cuff inflation period from 35 to 40 min were used as the values at each local skin temperature of sitting rest and at 40 min of passive heating and cuff inflation, respectively. In addition, we used the mean values of each local skin temperature that were measured during the cuff deflation protocol for 9 min as the values of the local skin temperatures at Pre and Post 60 (Figure 1).

### 2.7 Statistical analysis

Values are expressed as mean ± standard deviation. To compare the popliteal venous blood velocity, diameter, blood flow, and shear rate responses to passive heating and cuff inflation, two‐way analysis of variance (ANOVA) with repeated measures was applied under each condition (passive heating leg and cuff inflation leg) using time (baseline, the left leg without passive heating and the right leg with cuff inflation, and at 40 min of passive heating and cuff inflation) and condition as fixed factors. When interaction (condition × time) was significant, a post hoc analysis was performed using a paired *t*‐test and a Bonferroni test to compare conditions and time points, respectively. A paired *t*‐test was used to compare HR, SBP, DBP, and MAP between sitting rest and 40 min of passive heating and cuff inflation. To compare the local skin temperatures of the thigh, calf, and feet at sitting rest and at 40 min of passive heating and cuff inflation, two‐way ANOVA with repeated measures was applied under each condition (passive heating leg and cuff inflation leg), using time (sitting rest and 40 min of passive heating and cuff inflation) and condition as fixed factors. When interaction was significant, a post hoc analysis was performed using a paired *t*‐test to compare conditions and time points. A paired *t*‐test was used to compare HR, SBP, DBP, and MAP between Pre and Post 60. To compare the calf venous volume at 60 mmHg of cuff pressure, the calf venous compliance at 20 mmHg of cuff pressure, and the local skin temperatures of the thigh, calf, and feet in both the left and right legs between Pre and Post 60, two‐way ANOVA with repeated measures was applied under each condition (passive heating leg and cuff inflation leg) using time (Pre and Post 60) and condition as fixed factors. When interaction was significant, a post hoc analysis was performed using a paired *t*‐test to compare conditions and time points. Statistical significance was accepted at *p* < 0.05. All statistical analyses were performed using SPSS ver. 28 (IBM Corp., Armonk, NY).

## 3 RESULTS

### 3.1 Popliteal venous blood flow responses in the passive heating and cuff inflation legs

**FIGURE 2:**
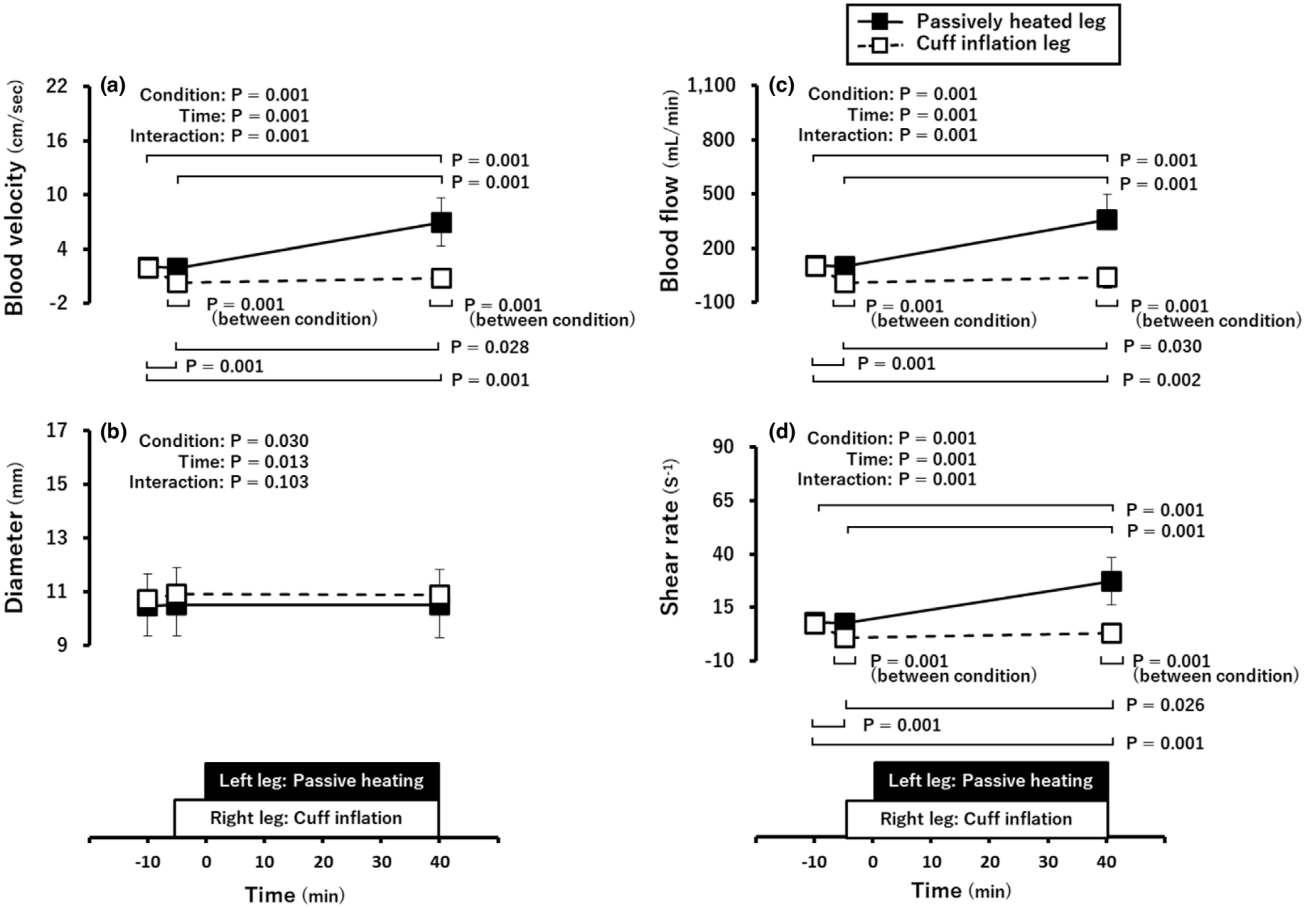
Popliteal venous blood velocity (a), diameter (b), blood flow (c), and shear rate (d) responses to passive heating and cuff inflation. Values are shown as the mean ± standard deviation.

Figure 2 shows the changes in blood velocity, diameter, blood flow, and shear rate of the popliteal vein in the left passive heating leg and right cuff inflation leg. There were no differences in the blood velocity, diameter, blood flow, or shear rate at baseline between the passive heating and cuff inflation legs (Figure 2a–d). The blood velocity in the left leg markedly increased with passive heating (*p* < 0.05), whereas the blood velocity in the right leg significantly decreased with cuff inflation, and this decrease was maintained throughout the protocol (Figure 2a). The blood velocity in the passive heating leg was greater than that in the cuff inflation leg at the end of the protocol (*p* < 0.05, Figure 2a). There was no significant interaction for the diameter (Figure 2b). The changes in blood flow and shear rate in the passive heating leg and cuff inflation leg were similar to the changes in the blood velocity (Figure 2c,d).

### 3.2 Changes in circulatory and body temperature parameters during passive heating and cuff inflation in sitting rest

HR increased with passive heating and cuff inflation (sitting rest, 70 ± 10 bpm; 40 min of passive heating and cuff inflation, 85 ± 11 bpm; *p* < 0.05). However, no changes were seen with passive heating and cuff inflation for 40 min in SBP (sitting rest, 110 ± 10 mmHg; 40 min of passive heating and cuff inflation, 108 ± 9 mmHg), DBP (sitting rest, 73 ± 7 mmHg; 40 min of passive heating and cuff inflation, 72 ± 6 mmHg), or MAP (sitting rest, 85 ± 7 mmHg; 40 min of passive heating and cuff inflation, 84 ± 7 mmHg).

At sitting rest, there was no difference in the local skin temperatures of the thigh, calf, and feet between the left and right legs. In the passive heating leg, the local skin temperatures of the calf (sitting rest, 32.0°C ± 0.6°C; 40 min of passive heating, 41.3°C ± 0.2°C; *p* < 0.05) and feet (sitting rest, 30.5°C ± 2.5°C; 40 min of passive heating, 41.1°C ± 0.2°C; *p* < 0.05) were significantly elevated, while that of the thigh fell with passive heating for 40 min (sitting rest, 32.3°C ± 0.8°C; 40 min of passive heating, 30.8°C ± 1.0°C; *p* < 0.05). In the cuff inflation leg, the local skin temperatures of the thigh (sitting rest, 32.2°C ± 0.7°C; 40 min of cuff inflation, 30.8°C ± 0.7°C; *p* < 0.05) and calf (sitting rest, 31.8°C ± 0.5°C; 40 min of cuff inflation, 30.4°C ± 0.7°C; *p* < 0.05) fell significantly, while that of the feet significantly elevated with cuff inflation for 40 min (sitting rest, 30.5°C ± 2.6°C; 40 min of cuff inflation, 31.9°C ± 2.8°C; *p* < 0.05). In addition, with 40 min of passive heating and cuff inflation, the local skin temperatures of the calf and feet were significantly higher in the passive heating leg than in the cuff inflation leg (*p* < 0.05), while that of the thigh did not differ between the two legs.

### 3.3 Calf venous properties, circulatory, and body temperature parameters at Pre and Post 60 in supine rest

**FIGURE 3:**
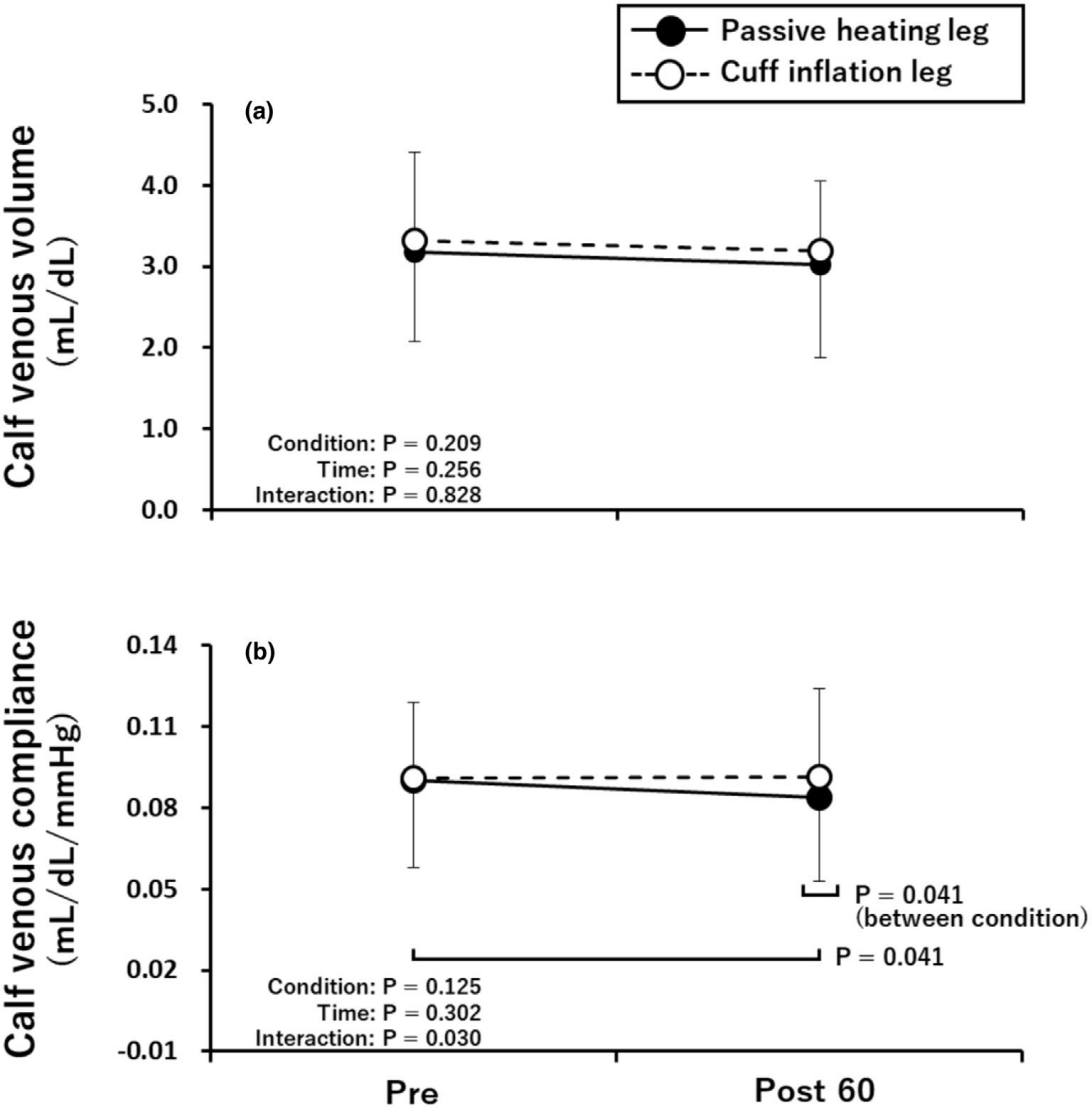
Effects of passive heating and cuff inflation on the venous volume (a) and venous compliance (b) in the calf. Pre, before passive heating and cuff inflation; Post 60, 60 min after passive heating and cuff inflation. Calf venous volume was assessed at 60 mmHg of cuff pressure. Calf venous compliance was assessed at 20 mmHg of cuff pressure. Values are shown as the mean ± standard deviation.

Figure 3 shows the effect of passive heating and cuff inflation on the venous volume and venous compliance in the calf. The calf venous volume in both legs did not differ between Pre and Post 60 (Figure 3a). In addition, while the calf venous compliance in the cuff inflation leg did not differ between Pre and Post 60, that in the passive heating leg was slightly but significantly lower at Post 60 than at Pre (*p* = 0.042). Moreover, calf venous compliance at Post 60 was lower in the passive heating leg than in the cuff inflation leg (*p* = 0.030, Figure 3b).

HR and BP were similar at Pre and Post 60 (Table 1). There were no differences in the local skin temperatures of the thigh, calf, and feet between the left and right legs at Pre. There was no significant interaction for local skin temperatures of the thigh (Table 2, *p* > 0.05). In the passive heating leg, the local skin temperatures of the calf and feet were significantly higher at Post 60 than at Pre (*p* < 0.05). In the cuff inflation leg, the local skin temperature of the calf was significantly lower at Post 60 than at Pre (*p* < 0.05) but not that of the feet between Pre and Post 60. In addition, the local skin temperatures of the calf and feet at Post 60 were significantly higher in the passive heating leg than in the cuff inflation leg (*p* < 0.05, Table 2).

**TABLE 1:** Circulatory parameters at Pre and Post 60.

	Pre	Post 60
Heart rate (bpm)	61 ± 8	60 ± 9
Systolic blood pressure (mmHg)	112 ± 10	114 ± 8
Diastolic blood pressure (mmHg)	68 ± 8	71 ± 6
Mean arterial blood pressure (mmHg)	83 ± 7	85 ± 5

*Note*: Values are shown as the mean ± standard deviation. Pre, before passive heating and cuff inflation; Post 60, 60 min after passive heating and cuff inflation.

**TABLE 2:** Body temperature parameters at Pre and Post 60.

	Pre	Post 60	Two‐way ANOVA		
			Condition	Time	Interaction
Thigh (°C)					
Passive heating leg	32.2 ± 0.8	31.5 ± 0.7	*p* = 0.001	*p* = 0.041	*p* = 0.072
Cuff inflation leg	32.0 ± 0.8	31.1 ± 0.8			
Calf (°C)					
Passive heating leg	32.4 ± 0.6	32.7 ± 0.5 *,***	*p* = 0.001	*p* = 0.001	*p* = 0.001
Cuff inflation leg	32.3 ± 0.6	31.0 ± 0.6 **			
Feet (°C)					
Passive heating leg	30.6 ± 2.6	32.3 ± 0.9 *,***	*p* = 0.001	*p* = 0.034	*p* = 0.003
Cuff inflation leg	30.5 ± 2.5	30.6 ± 2.1			

*Note*: Values are shown as the mean ± standard deviation. Pre, before passive heating and cuff inflation; Post 60, 60 min after passive heating and cuff inflation; ANOVA, analysis of variance.

* *p* < 0.05 versus Passive heating leg at Pre.

** *p* < 0.05 versus cuff inflation leg at Pre.

*** *p* < 0.05, Passive heating leg at Post 60 versus cuff inflation leg at Post 60.

## 4 DISCUSSION

### 4.1 New findings

The purpose of this study was to clarify the effects of acute passive heating and thigh cuff inflation on the volume and compliance in calf veins in healthy young adults. We obtained several new findings. First, in the passively heated left leg, calf venous compliance decreased, but calf venous volume did not change after passive heating compared with baseline, despite increases in blood flow and shear rate in the popliteal vein with passive heating. Second, following cuff inflation on the right leg, the volume and compliance of the calf veins did not change following cuff inflation that evokes decreased blood flow and shear rate in the popliteal veins. These findings suggest that acute passive heating decreases calf venous compliance with no change in calf venous volume, while acute thigh cuff inflation does not alter the volume and compliance in calf veins. To our knowledge, this is the first study to examine the acute effects of passive heating and thigh cuff inflation on the volume and compliance in calf veins in young healthy adults.

### 4.2 Effects of passive heating on the calf venous properties

Acute passive heating decreased the calf venous compliance without a change in calf venous volume (Figure 3), despite increases in blood flow and shear rate in the popliteal vein with passive heating. This finding did not support our hypothesis. Previous studies on arterial vessels have reported that the degree of shear rate increase with passive heating modulates endothelial function: endothelial function enhances at 5–6‐fold increases (Chaseling et al., 2023; Romero et al., 2017), while no change occurs at 2–3‐fold increases (Restaino et al., 2015; Teixeira et al., 2017). Furthermore, prolonged sitting rest that reduces shear rate is associated with decreased endothelial function (Thosar et al., 2015). These findings suggest that increased shear rate at least does not impair endothelial function, whereas decreased shear rate is linked to endothelial dysfunction. In contrast, in this study, passive heating decreased venous compliance with no change in venous volume despite increased venous shear rate by approximately 3.4‐fold (Figure 2d). The magnitude of increased shear rate in this study (approximately ~3.4‐fold) did not reach the 5–6‐fold level associated with improvement in arteries; rather, it was closer to the 2–3‐fold range reported to be associated with no change. Therefore, we cannot rule out that an ~3.4‐fold increase in shear rate was insufficient to stimulate venous endothelial function. However, it is unlikely that the increase in shear rate per se decreased venous compliance. Consequently, the reduced venous compliance with no change in venous volume is more likely attributable to factors other than the relatively modest increase in shear rate with passive heating.

Despite no change in calf venous volume, the reduction in calf venous compliance can be explained by the concept of volume and by the fact that the pressure–venous volume relationship is nonlinear. Volume equals the sum of stressed blood volume (SBV) and unstressed blood volume (UBV). SBV is the portion that generates transmural pressure and wall tension and is the principal component that exhibits immediate volume changes in response to pressure changes, whereas UBV is the volume filled at zero transmural pressure and is not the primary driver of immediate volume changes proportional to pressure (Magder, 2016; Persichini et al., 2022; Rothe, 1983). The venous occlusion plethysmography used to measure volume employs a relative Δvolume method that sets the initial value to zero. The cuff deflation protocol used in this study (Halliwill et al., 1999) increases volume (SBV + UBV) over 8 min of occlusion and then calculates compliance from Δvolume (principally ΔSBV)/Δcuff pressure during the subsequent deflation (Halliwill et al., 1999; Magder, 2016). From the change in cuff pressure and calf volume during deflation, a pressure‐venous volume relation can be derived. The cuff pressure‐venous volume relationship is nonlinear (i.e., curvilinear relationship), and Δvenous volume per unit Δcuff pressure is smaller in the high‐pressures range and larger in the low‐pressures range. The following discusses the possibility based on these concepts and their relationships.

In this study, calf skin temperature was elevated at Post 60 (Table 2), and because skin temperature reflects the skin blood flow response (Honda et al., 1963), we infer that, compared with Pre, blood pooling in calf veins (including cutaneous vessels) had already occurred before the measurement of volume change at Post 60 (i.e., a relative increase in UBV and a relative decrease in SBV). Accordingly, at the end of the 8 min inflation, overall calf venous volume was similar between Pre and Post 60, but SBV was relatively lower and UBV remained relatively elevated at Post 60 compared with Pre. In addition, during deflation, because the “stock” of SBV was smaller at Post 60 than at Pre, the same pressure change produced a smaller volume change at Post 60 than at Pre; in other words, calf venous compliance decreased at Post 60. Therefore, the change in balance between SBV and UBV after acute passive heating may induce the co‐existence of reduced calf venous compliance with no change in calf venous volume.

The observed decrease in calf venous compliance despite no change in calf venous volume may be contributing to circulatory homeostasis after passive heating. Since venous compliance equals Δvolume/Δpressure, the observed decrease in calf venous compliance indicates that it was more difficult for the calf veins to accumulate additional blood volume. In general, during passive heating, the limb veins provide venous capacitance that accommodates greater blood volumes in superficial vessels (Charkoudian, 2010; Cui et al., 2005; Maruyama et al., 2006), thereby lowering central blood volume and increasing HR (Crandall & González‐Alonso, 2010). During recovery, blood needs to be redistributed to the central circulation (Rowell, 1974).

If decreased vascular tone in the skin layer persists after passive heating, peripheral blood pooling will increase or be maintained, negatively affecting redistribution to central circulation (Charkoudian, 2010; Johnson & Kellogg, 2010). Thus, a transient adjustment that reduces venous compliance could limit excessive peripheral pooling and facilitate venous return (contributing to the redistribution of blood to the central circulation) during recovery.

### 4.3 Effects of thigh cuff inflation on the calf venous properties

Previous studies have reported that cuff inflation is likely to induce venous distension reflex that activates sympathetic nerve activity and raises BP and HR (Cui et al., 2011). However, in our study, BP did not increase during cuff inflation for 40 min. This appears to contradict previous findings, but differences in study design likely explain the discrepancy. In Cui et al. (2011), venous distension was induced when cuff inflation was combined with arterial ischemia and saline infusion, whereas we applied thigh cuff inflation at 80–110 mmHg alone. Consistent with this, Cui et al. (2015) reported no BP rise with thigh cuff inflation alone at 250 mmHg. Furthermore, reflex sympathetic responses return rapidly to baseline after the release of the stimulus (Cui et al., 2011; Williamson et al., 1994).

Therefore, we interpret the HR increase observed during the intervention as a thermoregulatory response to passive heating: passive heating raises core temperature, which activates cardiac sympathetic outflow (Crandall & González‐Alonso, 2010; Rowell, 1974). Although we did not measure core temperature, similar designs have documented increases in both core temperature and HR (Astrup et al., 1988; Gallen & Macdonald, 1990; Rodrigues et al., 2020). Accordingly, thigh cuff inflation in this study was unlikely to cause venous distension reflex, and the elevated HR during the intervention was likely attributed to passive heating rather than thigh cuff inflation.

In arteries, limb cuff inflation has been reported to reduce endothelial function and shear rate (Δ‐79 s^−1^; from 83 to 4 s^−1^) in a pressure‐dependent manner (Thijssen et al., 2009). Cuff inflation‐induced decrease in endothelial function is linked to increased oxidative stress (Hwang et al., 2003) and endothelin‐1 (Spieker et al., 2003), both of which can blunt endothelial function (Nishiyama et al., 2017). In our study, thigh cuff inflation similarly reduced popliteal venous shear rate (Δ‐4.3 ± 4.6 s^−1^; from 7.2 ± 2.8 s^−1^ to 3.0 ± 3.9 s^−1^) (Figure 2d). Additionally, cuff inflation can also raise circulating vasoconstrictors such as ET‐1 and angiotensin II (Colombo et al., 2014; Lin et al., 2017). Nevertheless, we observed no change in calf venous volume or compliance after thigh cuff inflation (Figure 3). Although we did not measure vasoconstrictor and therefore cannot determine the mechanism directly, we speculate that reactive hyperemia immediately after cuff release promoted venous dilation and counterbalanced any vasoconstrictor influence of cuff inflation‐induced congestion. Indeed, the cross‐sectional area of forearm superficial (Mollison et al., 2006) and popliteal (Libertiny & Hands, 1999) veins increases during reactive hyperemia, and the diameter changes observed during reactive hyperemia can be abolished when peripheral venous outflow is impeded (Libertiny & Hands, 1999). Taken together, opposing post‐deflation vasodilatory and inflation‐phase constrictor effects may have yielded a net no change in calf venous volume and compliance.

Thosar et al. (2015) reported that arterial shear rate and endothelial function decline after approximately 3 h of sitting. Indeed, in this study, subjects maintained approximately 3 h of rest (supine and seated) throughout the protocol (Figure 1). Furthermore, in the cuff inflation leg, local skin temperature of the calf also decreased (Table 2). This skin temperature change speculates a decrease in skin blood flow (Honda et al., 1963). However, no changes were observed in the volume and compliance in calf veins (Figure 3). This result contradicted the findings reported by Thosar et al. (2015). The discrepancy between previous studies and our findings may stem from differences in posture in order to evaluate vascular properties. Specifically, previous studies assessed vascular function while maintaining the seated position after the seated rest intervention. In contrast, our study involved performing passive heating and cuff inflation in the seated position, followed by a transition to the supine position for vascular property evaluation. In addition, the slight muscle contraction that occurs during the transition from the seated resting position to the supine position may increase blood flow and shear rate, potentially abolishing the decline in endothelial function caused by prolonged seated rest (Padilla et al., 2009; Thosar et al., 2015). Furthermore, it has been reported that arterial blood flow and shear rate in the lower limbs are higher in the supine position compared to the seated position (Newcomer et al., 2008). Based on these prior studies, it is conceivable that the slight transition from sitting to the supine position in this study, along with the accompanying improvements in blood flow and shear stress, may have counteracted the effects of prolonged rest (physical inactivity).

### 4.4 Comparison of effects on passive heating and thigh cuff inflation

In our study, despite reductions in venous blood flow and shear rate during thigh cuff inflation, the volume and compliance in calf veins were unchanged; by contrast, during the passive heating intervention, venous blood flow and shear rate increased, calf venous compliance decreased, and calf venous volume remained unchanged. At first glance, these results appear to suggest that the increase in shear rate with passive heating is detrimental to venous compliance. Indeed, applying arterial‐level blood flow and shear rate to venous segments induces harmful remodeling (thickening of the intima and media) (Casey et al., 2001; Miyakawa et al., 2008). However, previous studies have shown that each vascular bed (arteries, veins, lymphatics) has a set point for the magnitude of shear stress, and that above the physiological range activates inflammatory pathways and suppresses the stabilization pathways (Baeyens et al., 2016). The wall shear rates occurring within the physiological range of veins can reach approximately ~6 dynes cm^−2^ (~170 s^−1^) (Park et al., 2019; Roux et al., 2020). The degree of increased shear rate in this study was 19.1 ± 10.8 s^−1^ (from 8.1 ± 3.7 s^−1^ to 27.2 ± 11.1 s^−1^; during 40 min of passive heating) (Figure 2d), which was within the physiological range. Therefore, it is unlikely that the increase in venous shear rate observed in this study adversely affected vascular compliance.

### 4.5 Perspective and significance

Arterial studies show that acute passive heating increases endothelial function and decreases vascular stiffness (Brunt, Jeckell, et al., 2016; Caldwell et al., 2017; Ogoh et al., 2016; Restaino et al., 2015; Sugawara & Tomoto, 2021; Teixeira et al., 2017), whereas cuff inflation reduces endothelial function (Thijssen et al., 2009). In contrast, our study showed that venous properties differed: passive heating reduced calf venous compliance without a change in calf venous volume, and cuff inflation does not alter the volume and compliance in calf veins. This disagreement suggests that arteries and veins may differ fundamentally in their responses to the same stimuli (heating and cuff inflation). Regarding the decrease in venous compliance with no change in venous volume after passive heating, we speculate that there is a change in the balance of UBV and SBV. By contrast, although we cannot identify a single reason for the absence of change after cuff inflation, veins are known to have lower sensitivity to vasoactive substances and lower endothelial production of such mediators than arteries (Zhang et al., 2004, 2006). Taken together, these considerations indicate that arterial findings should not be directly extrapolated to veins and underscore the need for further investigations that assume vein‐specific mechanisms.

Very little data have been published evaluating the effects of passive heating on calf venous compliance. Maruyama et al. (2006) have reported that calf venous compliance increased with repeated heat exposure (40°C, 40%RH; 4 h/day, 9–10 days of heat exposure). Although Maruyama and colleagues did not assess blood flow or shear rate, they interpreted the increase in calf venous compliance as being attributable to greater NO bioavailability secondary to heat‐stress–induced increases in blood flow and shear. In addition, repeated blood‐flow and shear stimuli for several weeks can elevate circulating NO metabolites (Wen et al., 2015). These studies suggest that prolonged passive heating may increase volume and compliance in calf veins. However, it is unknown whether repeated acute local passive heating increases the volume and compliance in calf veins. Conversely, prolonged sitting leads to dependent venous congestion and reduced venous blood flow (Hitos et al., 2007), and prolonged seated immobility is associated with an increased risk of venous thromboembolism (Eberhardt & Raffetto, 2014). Moreover, thrombus‐related venous obstruction and remodeling in chronic venous disease are associated with reduced venous distensibility (Skoog et al., 2020; Turner et al., 2000). These studies suggest that chronic venous congestion may reduce venous compliance; however, it also remains unclear whether repeated venous congestion induced by cuff inflation would decrease venous properties. Therefore, to clarify the effect of both increases and decreases in blood flow and shear rate in veins on the venous properties, it will be necessary to examine the long‐term effects of passive heating and cuff inflation. This future investigation is expected to inform strategies for hypertension prevention and contribute to the maintenance and improvement of cardiovascular health.

### 4.6 Limitations

This study has some limitations. First, calf volume measured by the VOP technique provides the vascular responses of all arteries, veins, and capillaries in the whole limb. Second, we used venous collecting cuff pressure as a surrogate for intravenous pressure. Nonetheless, as previously described (Monahan et al., 2001), we believe that this assumption is appropriate (Halliwill et al., 1999). Third, because it was difficult to evaluate the blood flow velocity in the popliteal vein from the cross‐sectional images of the vessel, we measured it using longitudinal images. Finally, this study involved young healthy participants, and we cannot extrapolate our findings to older individuals or those with cardiovascular disease. However, these limitations do not directly affect the results of the present study.

## 5 CONCLUSION

This study investigated the acute effects of passive heating and thigh cuff inflation on the volume and compliance in calf veins. The primary findings were that passive heating decreases calf venous compliance without a change in calf venous volume, whereas thigh cuff inflation does not alter the volume and compliance in calf veins in healthy young adults. The change in venous properties after passive heating is likely to contribute to a recovery‐phase adjustment that helps limit excessive peripheral pooling and facilitate venous return. In addition, compared with passive heating, the effects of thigh cuff inflation (80–110 mmHg) on calf venous properties may be more readily masked by minor body movements or by rapid cuff deflation.

## AUTHOR CONTRIBUTIONS

YI and AO, conception and design of the work. YI and AO, conducted experiments. YI, acquisition, analysis, or interpretation of data for the work. YI and AO, drafting the work or revising it critically for important intellectual content. All authors read and approved the manuscript.

## FUNDING INFORMATION

This research was supported by a grant from the Japan Society for the Promotion of Science, Grant/Award Number: 18K10974.

## CONFLICT OF INTEREST STATEMENT

The authors have no financial conflicts of interest to declare.

## Data Availability

The data presented in this study is available on request from the corresponding author due to ethical reasons.
